# Amorphous solid dispersions as a strategy to enhance the bioavailability and stability of formulations containing plant active ingredients: An integrative review

**DOI:** 10.1007/s40199-026-00594-1

**Published:** 2026-03-09

**Authors:** Maria Eduarda de Oliveira Cardoso Melo, Camila Castro da Silva, Thainá dos Santos Dantas, Janaina Carla Barbosa Machado, Mágda Rhayanny Assunção Ferreira, Luiz Alberto Lira Soares

**Affiliations:** Federal University of Pernambuco, Recife, Brazil

**Keywords:** Herbal medicine, Phytopharmaceuticals, Drug delivery, Polymer carriers, Bioavailability

## Abstract

**Objectives:**

Plant-derived active pharmaceutical ingredients (APIs), including crude extracts, phytocomplexes, and isolated compounds, have been extensively investigated due to their therapeutic potential. However, their pharmaceutical application is often hindered by challenges such as low solubility, limited stability, and poor bioavailability. Amorphous solid dispersions (ASDs) have emerged as a promising strategy to overcome these limitations, enhancing solubility, dissolution, and oral absorption of these compounds, while also protecting active constituents and improving their stability. Accordingly, this review aims to explore the use of ASDs for plant-derived APIs, focusing on production methods, polymers and carriers employed, as well as characterization techniques applied to these formulations.

**Methods:**

A literature search was conducted in ScienceDirect, Scopus, Web of Science and PubMed databases for articles published between 2000 and 2025. Using terms related to ASDs and plant-derived compounds, 566 articles were retrieved, of which 84 met the inclusion criteria after screening.

**Results and discussion:**

The review highlights successful incorporation of several plant-derived APIs into ASDs, primarily to improve solubility and membrane permeability, with isolated compounds being the most frequently studied. The solvent evaporation method was the most used, although efforts toward more sustainable production methods were also reported. Among polymeric carriers, poly(vinylpyrrolidone) (PVP), poly(vinyl alcohol) (PVA), and poly(ethylene glycol) (PEG) were the most frequently employed. The most common physicochemical characterization techniques included scanning electron microscopy (SEM), Fourier transform infrared spectroscopy (FTIR), differential scanning calorimetry (DSC), and X-ray diffraction (XRD).

**Conclusion:**

Overall, ASDs represent a viable and effective approach to unlock the therapeutic potential of plant-derived APIs, supporting the development of more stable and bioactive herbal formulations.

## Introduction

Plant-derived active pharmaceutical ingredients (APIs) encompass a broad range of bioactive materials of botanical origin, including crude extracts and tinctures (crude APIs) as well as fractions and isolated compounds (isolated APIs), such as flavonoids and tannins. These botanical derivatives have historically been used in healthcare, both in traditional medicine and in industrialized formulations [1–3]. They are associated with diverse pharmacological activities, including antioxidant, anti-inflammatory, antimicrobial, and anticancer effects, which supports their continued relevance as therapeutic options and as sources of lead compounds for drug development [3, 4]. In parallel, interest in plant-derived therapeutics has expanded, driven by the renewed integration of traditional herbal medicine with modern drug-discovery and clinical development efforts and by consumer preference for products perceived as effective and better tolerated than conventional synthetic drugs [5–7].

However, increased interest does not necessarily translate into consistent pharmaceutical performance. Key biopharmaceutical and stability limitations, particularly low solubility, dissolution and susceptibility to degradation or solid-state transformations, often hinder the development of reproducible plant-derived products [8, 9]. Crude APIs are typically complex multicomponent systems that may exhibit instability under gastrointestinal conditions, low membrane permeability, and poor solubility, including the formation of poorly soluble aggregates or complexes driven by intermolecular interactions [10]. For isolated APIs, extraction, isolation and purification from complex matrices may result in low yields, and many isolated constituents still display restricted solubility, frequently requiring organic solvents during upstream processing, which can further complicate pharmaceutical development and translation [11]. Overall, both crude and isolated plant-derived APIs are prone to instability during manufacturing and storage and may undergo degradation after administration, collectively compromising bioavailability and shelf-life.

To overcome these limitations, amorphous solid dispersions (ASDs) have emerged as an effective formulation strategy to enhance the apparent solubility and oral bioavailability of poorly soluble compounds, thereby improving dissolution, absorption, and the likelihood of achieving therapeutic exposure [12–16]. In ASDs, the API is dispersed, ideally at the molecular level, within a polymeric carrier, and the amorphous state increases the system’s free energy, which can accelerate dissolution and promote supersaturation in gastrointestinal fluids [17–20]. Beyond solubility enhancement, ASD approaches may also improve downstream processability such as powder flow and compressibility, which is relevant for scale-up and consistent pharmaceutical quality [21, 22].

Therefore, given these technological challenges, this review examines the application of amorphous solid dispersions (ASDs) to plant-derived active pharmaceutical ingredients, with emphasis on production methods, polymeric carriers, and characterization techniques. The aim is to evaluate whether ASDs can overcome limitations of solubility, bioavailability, and stability, while outlining perspectives for the future development of phytopharmaceutical formulations.

Although ASDs have been extensively reviewed as a platform to improve the oral performance of poorly soluble small-molecule drugs, guidance tailored to plant-derived APIs remains comparatively fragmented [20, 21, 23]. Existing reviews often address solid dispersions across broad classes of molecules without focusing on the distinctive challenges posed by botanical materials, whereas studies specifically discussing ASDs derived from herbal extracts frequently emphasize individual case examples rather than providing a unified development framework. Consequently, an integrative synthesis that links the compositional complexity of plant-derived APIs (from multicomponent extracts to isolated phytochemicals), their characteristic biopharmaceutical and solid-state/stability liabilities, and formulation design choices, polymeric carriers, manufacturing routes, and fit-for-purpose characterization workflows, needed to obtain reproducible solid dosage forms remains limited.

In this context, this integrative review examines the application of ASDs to plant-derived APIs, emphasizing preparation methods, polymeric carriers, and fit-for-purpose characterization of both performance and stability. In addition, the review consolidates the current evidence and outlines perspectives and practical considerations to support the future development of phytopharmaceutical solid formulations.

## Methodology

This integrative review was conducted through a structured literature search in ScienceDirect, PubMed, Scopus, and Web of Science. These databases were selected to provide complementary coverage across pharmaceutics/formulation and biomedical literature. To mitigate the risk of missing relevant studies, the reference lists of all included articles were manually screened.

### Search strategy

Database-specific search strings were developed based on three concept blocks: solid dispersions/ASDs, plant-derived APIs, and performance endpoints (solubility, bioavailability, and stability). The strategy was adapted to the syntax of each database. In ScienceDirect, the search query was: “Amorphous Solid Dispersions” AND (“plant extract” OR plant OR vegetal OR “botanical extract” OR herb) AND (bioavailability OR solubility OR stability). In PubMed, the search terms were: (“solid dispersion” OR “amorphous solid dispersion” OR ASD OR ASDs) AND (“plant extract” OR “botanical” OR “herbal” OR “phytochemical” OR “plant-derived”) AND (solubility OR bioavailability OR stability). In Scopus and Web of Science, equivalent combinations of these terms were applied using the respective advanced-search fields (TITLE-ABS-KEY in Scopus; TS in Web of Science). Searches were limited to publications in English or Portuguese between 2000 and 2025. When available, filters were applied to restrict results to original research articles.

### Eligibility criteria

Studies were eligible if they met all the following inclusion criteria: were original research articles (experimental studies); investigated solid dispersions and/or amorphous solid dispersions (ASDs) as the main formulation strategy; used plant-derived APIs as the central component of the formulation, including multicomponent extracts, enriched fractions, or isolated phytochemicals; and reported at least one outcome relevant to the scope of this review; solubility/dissolution, bioavailability or absorption-related performance (e.g., in vivo pharmacokinetics, ex vivo permeability, or validated surrogate performance assays), and/or physical/chemical stability, together with adequate formulation and solid-state characterization (e.g., XRPD, DSC, FTIR/Raman, microscopy, or equivalent methods).

### Exclusion criteria

The following were excluded: review articles, editorials, letters, conference abstracts, theses/dissertations, patents, and book chapters; studies not focused on plant-derived APIs (e.g., synthetic drugs only); studies in which solid dispersions were not the primary formulation approach; articles without sufficient methodological detail or without extractable data relevant to the outcomes of interest; and papers for which the full text could not be accessed for eligibility assessment.

### Study selection and screening

Records retrieved from the databases were collated and screened in three stages. First by title/abstract screening, following for full-text assessment, and final inclusion. Two reviewers independently screened titles/abstracts and full texts according to the predefined eligibility criteria; disagreements were resolved by consensus.

### Data extraction and synthesis

Data were extracted using a standardized form capturing type of plant-derived API (extract/fraction/isolated compound), carrier polymer(s), manufacturing method, key solid-state and physicochemical characterization techniques, and reported effects on solubility/dissolution, bioavailability-related performance, and stability. Given the heterogeneity of experimental designs and outcome measures, findings were synthesized narratively, with emphasis on formulation design choices and considerations relevant to reproducible development.

**Fig. 1:**
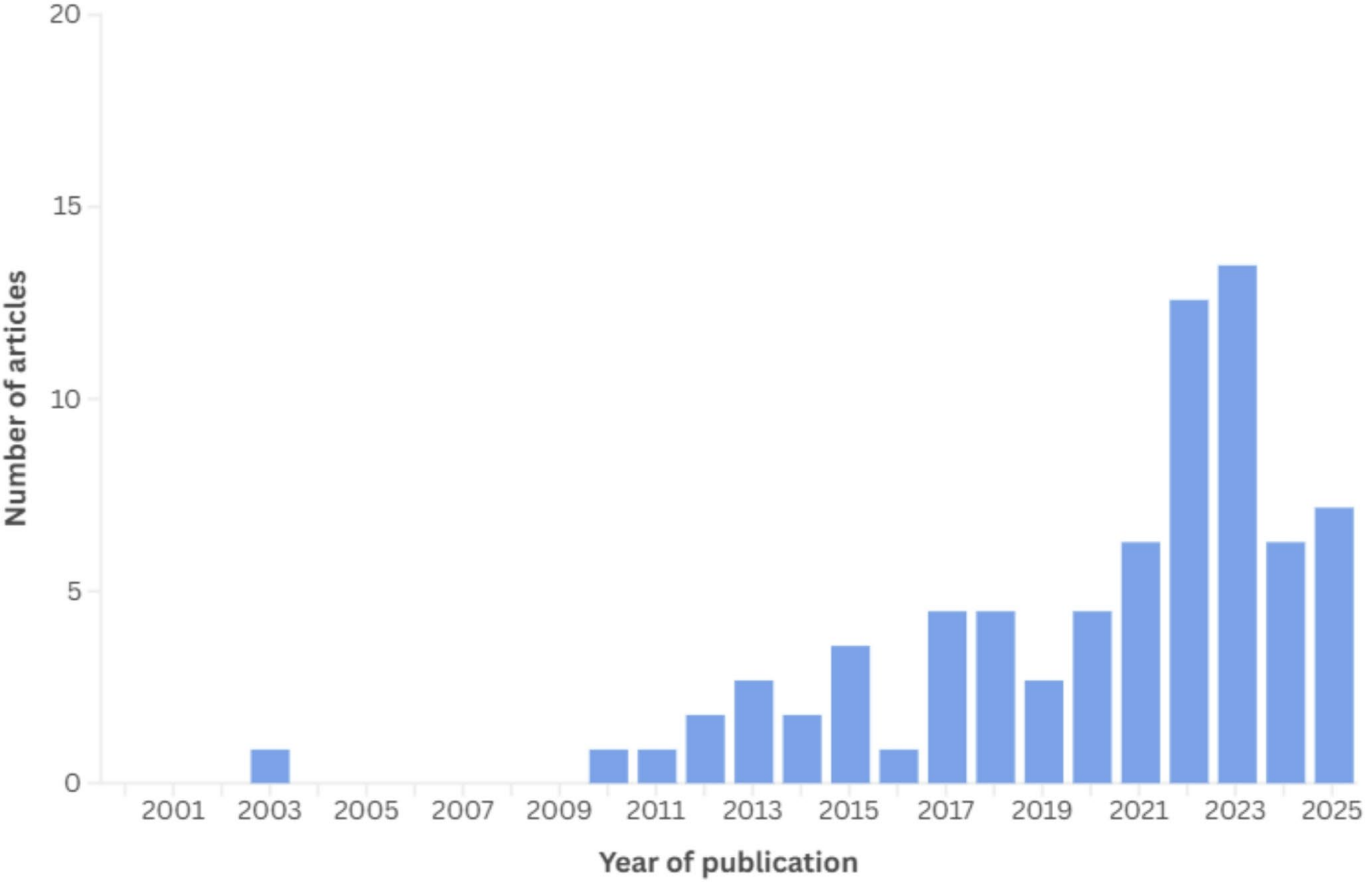
Distribution of articles by year of publication

**Fig. 2:**
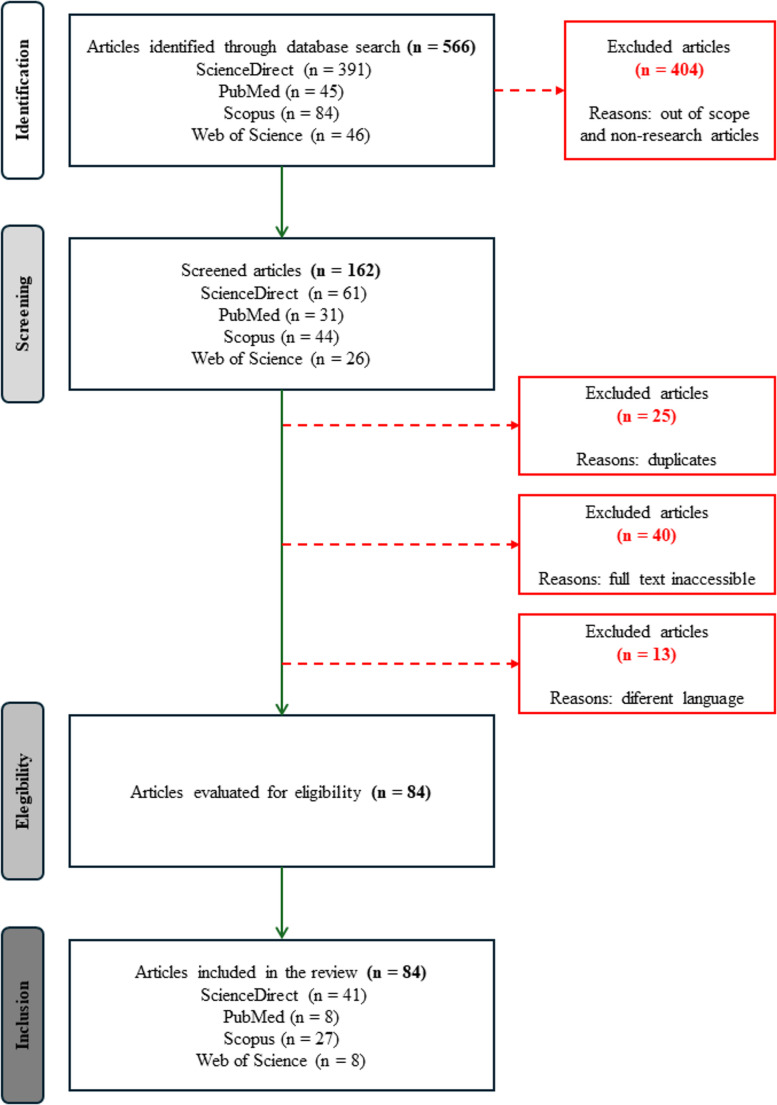
Flowchart of the search and selection strategy of the articles

The distribution of the articles by years and the flowchart of the searching flow are presented in the Figs. 1 and 2, respectively.

## Results

### Characteristics and limitations of plant-derived active pharmaceutical ingredients (APIs)

Plant-derived active pharmaceutical ingredients (APIs) range from extracts and other derivatives to isolated compounds obtained from botanical species, and they have been widely used in traditional medicine. In recent years, interest in incorporating these substances into phytopharmaceutical formulations has grown significantly, driven by the broad spectrum of biological activities exhibited by phytochemicals [24]. These include antioxidant, anti-inflammatory, anticancer, and neuroprotective effects, among others.

However, the low aqueous solubility characteristic of many of these bioactive compounds represents a major limitation for their therapeutic application [25, 26]. As a result, oral absorption is often compromised, leading to reduced therapeutic efficacy. To overcome this challenge, amorphization has emerged as a promising strategy to improve the solubility and bioavailability of plant-derived APIs [25].

To date, relatively few studies have reported the incorporation of crude plant-derived APIs into amorphous solid dispersions (ASDs), whereas isolated compounds have been more extensively investigated. Table 1 summarizes the main specificities that highlight the need for amorphization of these ingredients.

**Table 1:**
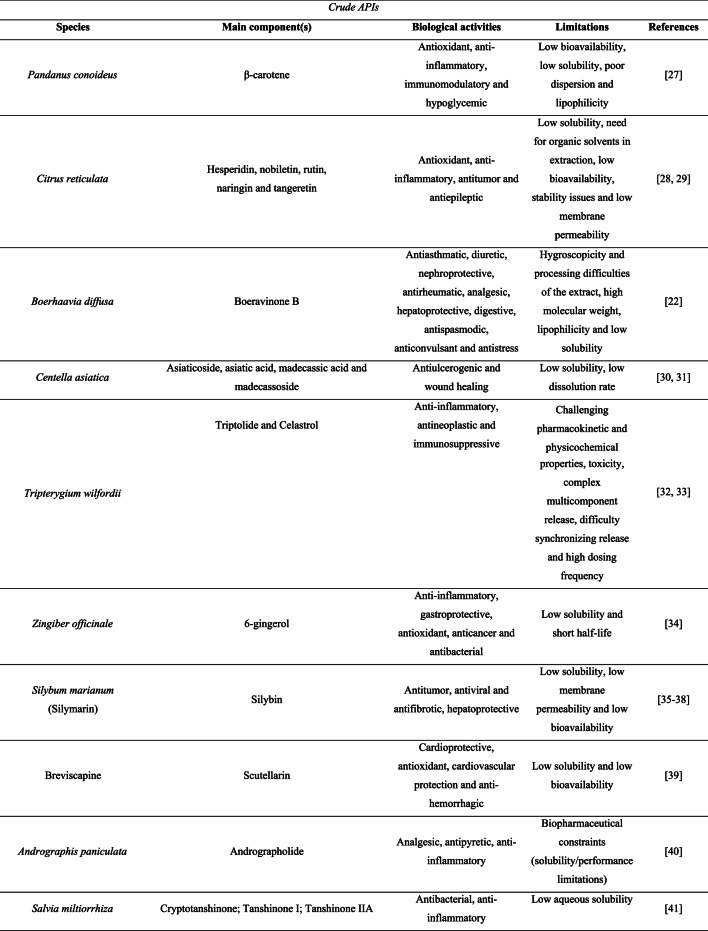

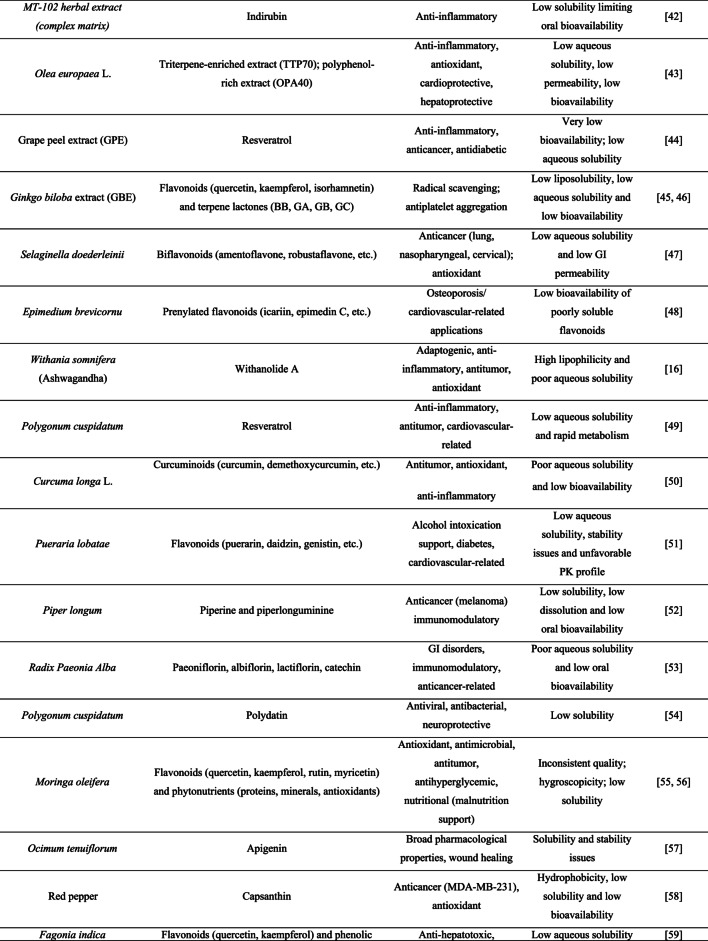

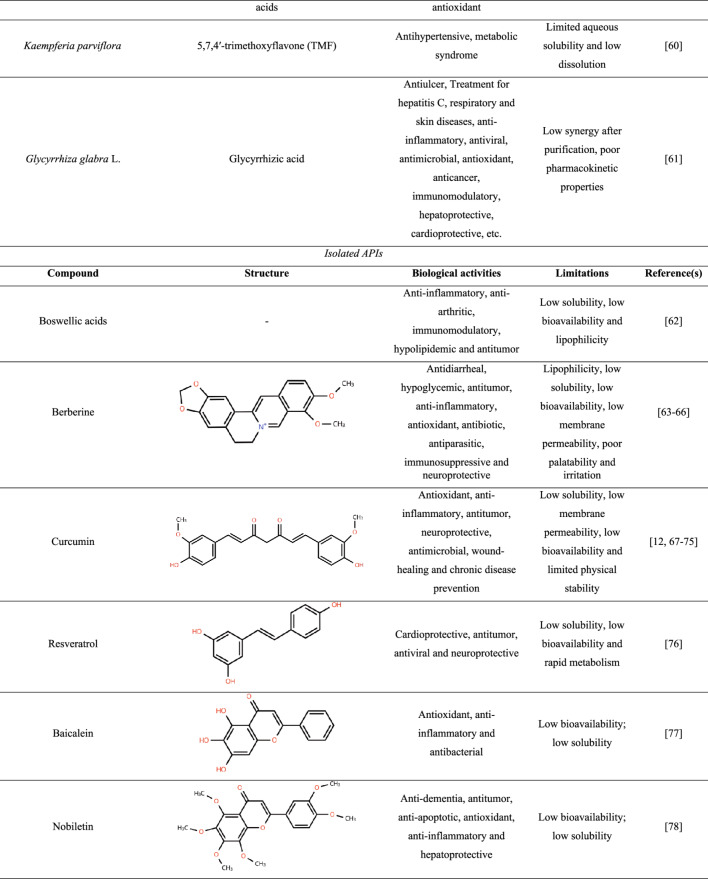

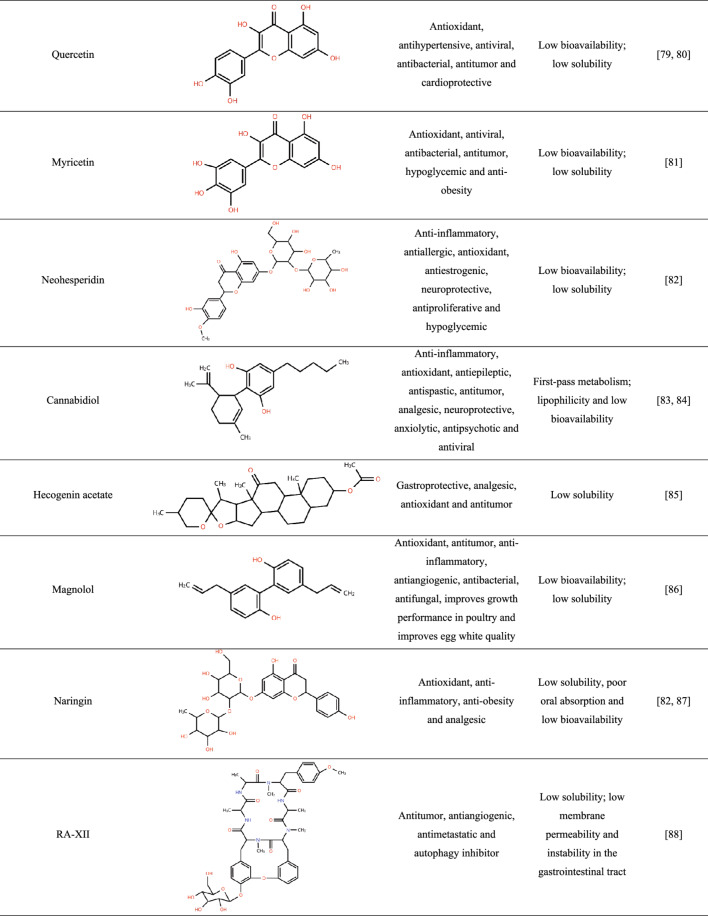

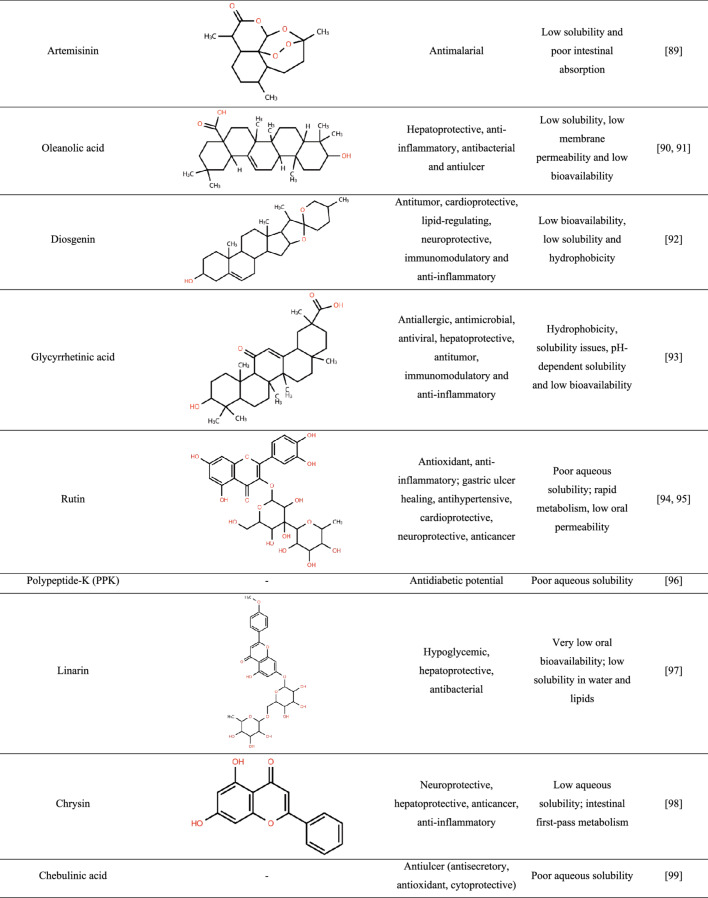

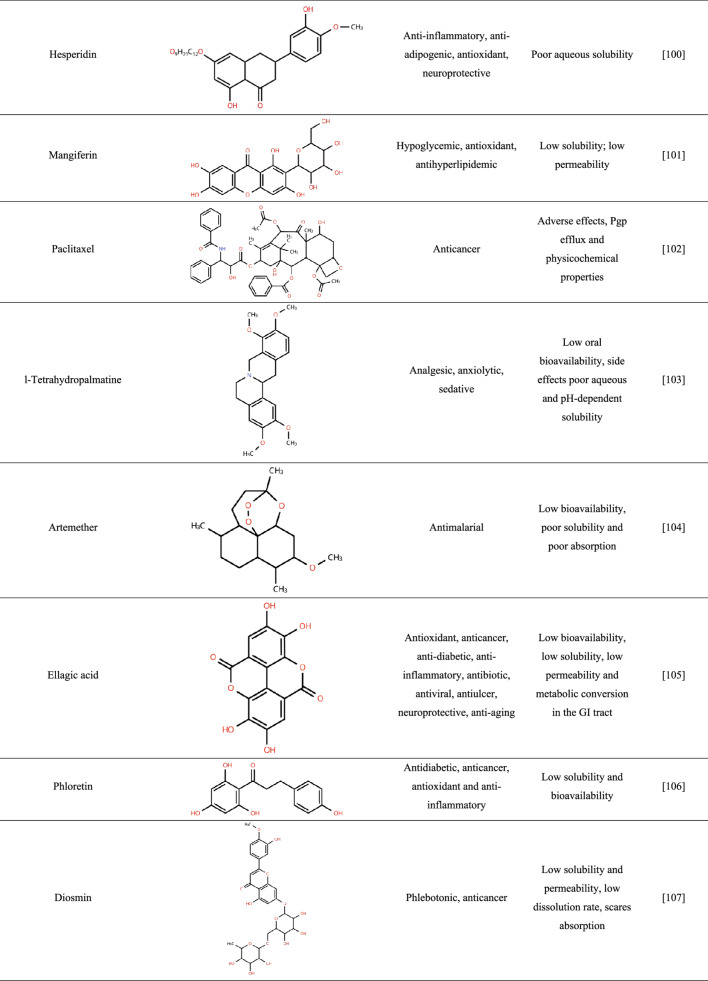
Features of plant-derived apis reported in the literature

### Production of amorphous solid dispersions (ASDs)

Amorphous solid dispersions (ASDs) are systems in which the active compound of interest is molecularly dispersed in a hydrophilic polymeric matrix in a disordered state. Their use has been widely described in the literature as a promising strategy to improve the bioavailability of poorly water-soluble compounds, particularly those classified as Class II under the Biopharmaceutics Classification System (BCS), by dispersing them in the amorphous form within a hydrophilic polymer matrix [17]. In addition to thermodynamically enhancing solubility, ASDs can generate metastable supersaturated solutions, which result in increased transport of the active compound across the gastrointestinal epithelial membrane. This advantage is not typically observed with other solubility-enhancing strategies, such as micellar solubilization or cyclodextrin complexation [108].

**Fig. 3:**
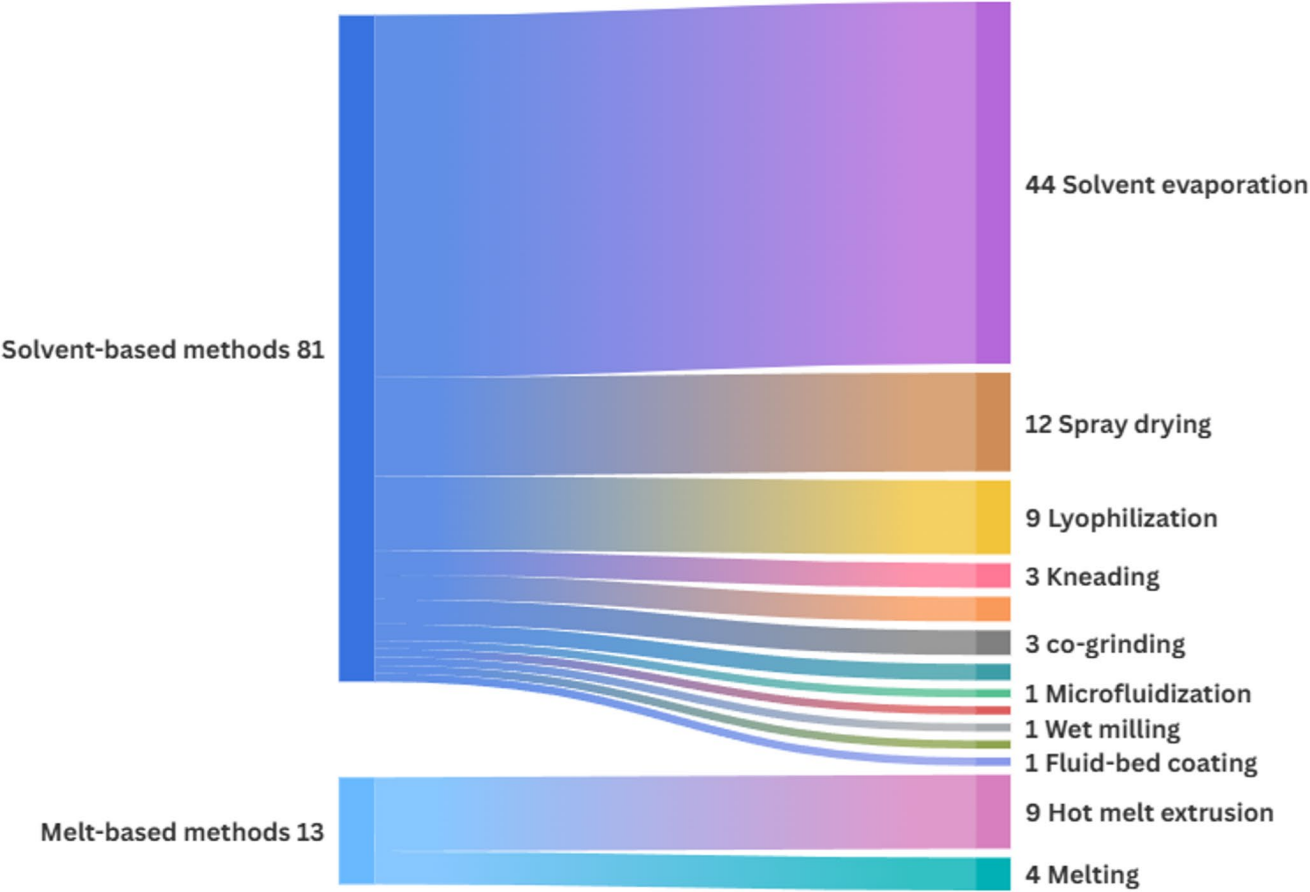
Chart representing the production methods used for plant-derived APIs

The production of ASDs can be achieved through two main approaches: melt-based methods and solvent-based methods [17]. Each technique presents specific advantages and limitations that directly influence the characteristics of the final product. Therefore, the selection of an appropriate method must consider multiple factors, including the physicochemical properties of both the active compound and the carriers, the desired release profile, cost, and scalability. Figure 3 illustrates the production methods employed in the literature for plant-derived APIs.

As shown in the flowchart (Fig. 3), ASDs involving plant-derived APIs have been predominantly produced using solvent-based methods, with solvent evaporation being the most frequently employed. This technique is widely used due to its simplicity and relatively low cost; however, it also presents disadvantages, such as the potential presence of residual organic solvents and the risk of phase separation during drying [109, 110]. The methods and their advantages and disadvantages are summarized below (Table 2).

**Table 2:** Advantages and disadvantages of manufacturing methods reported in the literature

Solvent-based methods			
Method	**Advantages**	**Disadvantages**	**References**
Solvent evaporation	Simple, low cost	Organic solvent residue, risk of solvent phase separation	[109, 110]
Spray drying	Requires a smaller amount of material, relatively low cost, can be applied to compounds with high melting points, allows easy control of particle size, versatile regarding the products used, enables controllable continuous processing, scalable, can induce amorphization at higher drug loads	Thermal degradation, organic solvent residue, loss of material (adhesion to the walls of the drying chamber), sensitivity to changes in flow rate, scalability challenges (modifications in droplet trajectory, evaporation rate, drying time)	[111–114]
Lyophilization	Preserves fragile structures, useful for heat-sensitive drugs	High cost, long processing time, not suitable for water-insoluble polymers, risk of collapse of the amorphous structure	[115, 116]
Spray freeze drying	High yield, smaller particle size, reduced stress on raw materials, porous product (high surface area), control over particle size	Limitation in the solvent used (low compatibility with organic solvents), produces porous and low-density dispersions that may be structurally fragile	[117]
Co-precipitation	Production of homogeneous particles, possible complete amorphization of the active compound	Use of organic solvents, difficult removal of residual solvents	[115]
Jet milling	Continuous process, no use of solvents, significant particle size reduction	High energy (may generate heat and degradation), instability of the amorphous state, possible processing losses	[118, 119]
Co-grinding	Green method, high yield, no need for solvents.	May cause high mechanical stress, difficulties in achieving homogeneity on a large scale.	[120]
Wet milling	Useful for high-moisture drugs, convenient, good scalability, simple, low cost	Limited particle size reduction	[121]
Kneading	Simple method, low cost, solvent-free, easy scalability, high yield	-	[122, 123]
Microfluidization	Particle size reduction, particle homogeneity, continuous manufacturing	High-cost equipment, rarely reported	[124]
Microwaves	Fast, low energy consumption	Experimental technique, limited industrial application	[115]
*Melt-based methods*			
Method	**Advantages**	**Disadvantages**	**References**
Hot melt extrusion (HME)	Solvent-free process, continuous production, can produce immediate- or controlled-release solid dosage forms, reduced production time, good scalability, improved powder flow	High initial, operational, and maintenance costs; sensitive to temperature variations; complex process optimization; limited suitability for thermosensitive actives; residual crystallization	[114, 125–127]
Melting	Solvent-free, low cost, simple method	High temperatures may cause thermal degradation; polymers require low glass transition temperature	[116, 117]

## Polymers and carriers

The polymers and carriers employed in the production of ASDs play a fundamental role in determining the characteristics and overall performance of the final dispersion. The effectiveness of ASDs largely depends on the interactions between the active compound and the selected polymeric carriers, which must be biocompatible, non-toxic, and capable of forming a stable dispersion that prevents recrystallization of the active ingredient [108]. While appropriate polymers can enhance the wettability of plant-derived APIs, improving dissolution and inhibiting precipitation, an inadequate choice may impair molecular mobility and reduce the chemical potential of the dispersed molecules [76].

Moreover, the ideal properties of the polymer depend on both the specific active ingredient and the production method employed. Factors such as glass transition temperature (Tg), degree of hydrophilicity, molecular weight, and chemical structure must be considered to ensure favorable interactions with plant-derived APIs and adequate resistance to processing conditions. The polymers most frequently employed in ASDs containing plant-derived APIs, as well as their frequency of use in the reviewed articles, are summarized in Table 3.

**Table 3:**
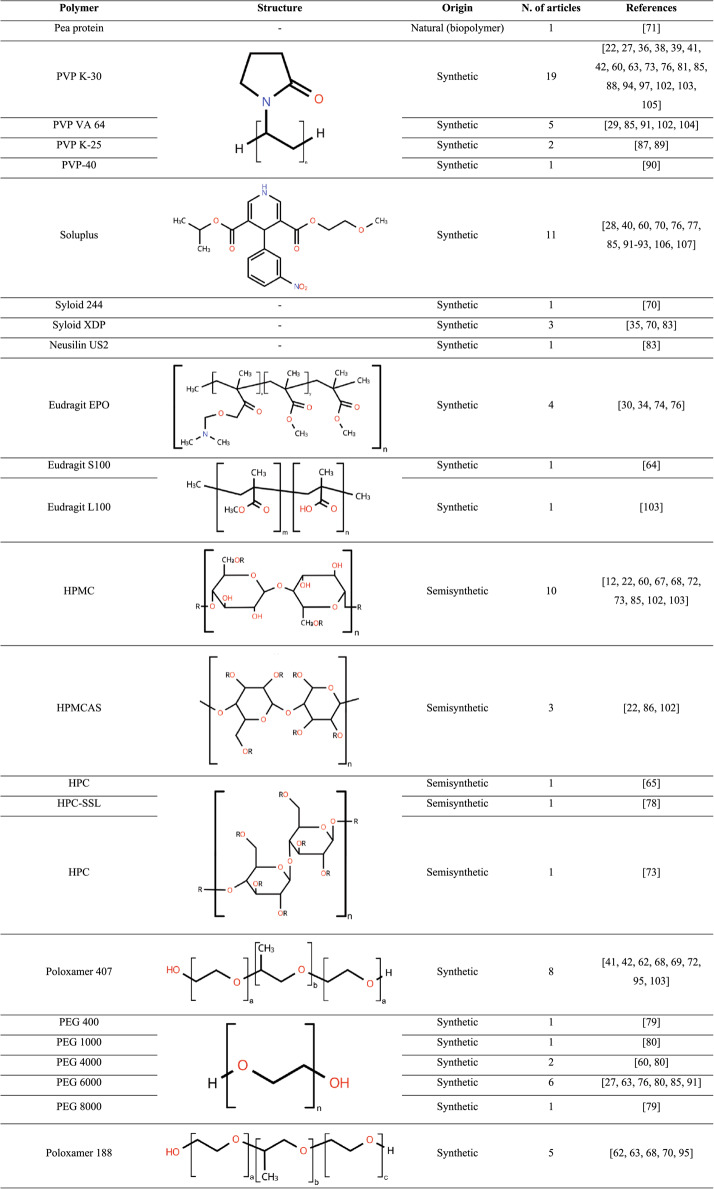

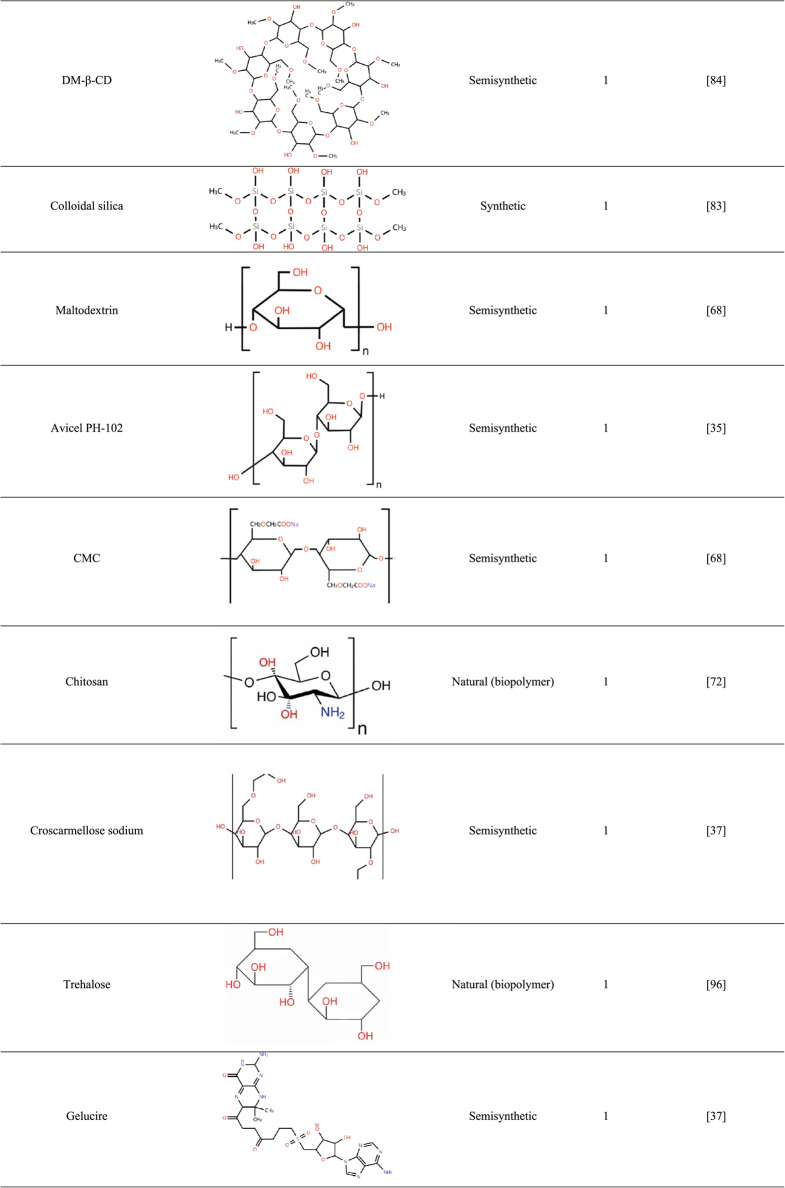
Characteristics and frequency of polymers and carriers employed

*PVP* poly(vinylpyrrolidone), *PEG* poly(ethylene glycol), *CMC* carboxymethyl cellulose, *HPMC* hydroxypropyl methylcellulose, *HPC* hydrogenated phosphatidylcholine, *DM*-β-*CD* dimethyl-beta-cyclodextrin

## Physicochemical characterization techniques of amorphous solid dispersions (ASDS)

**Fig. 4:**
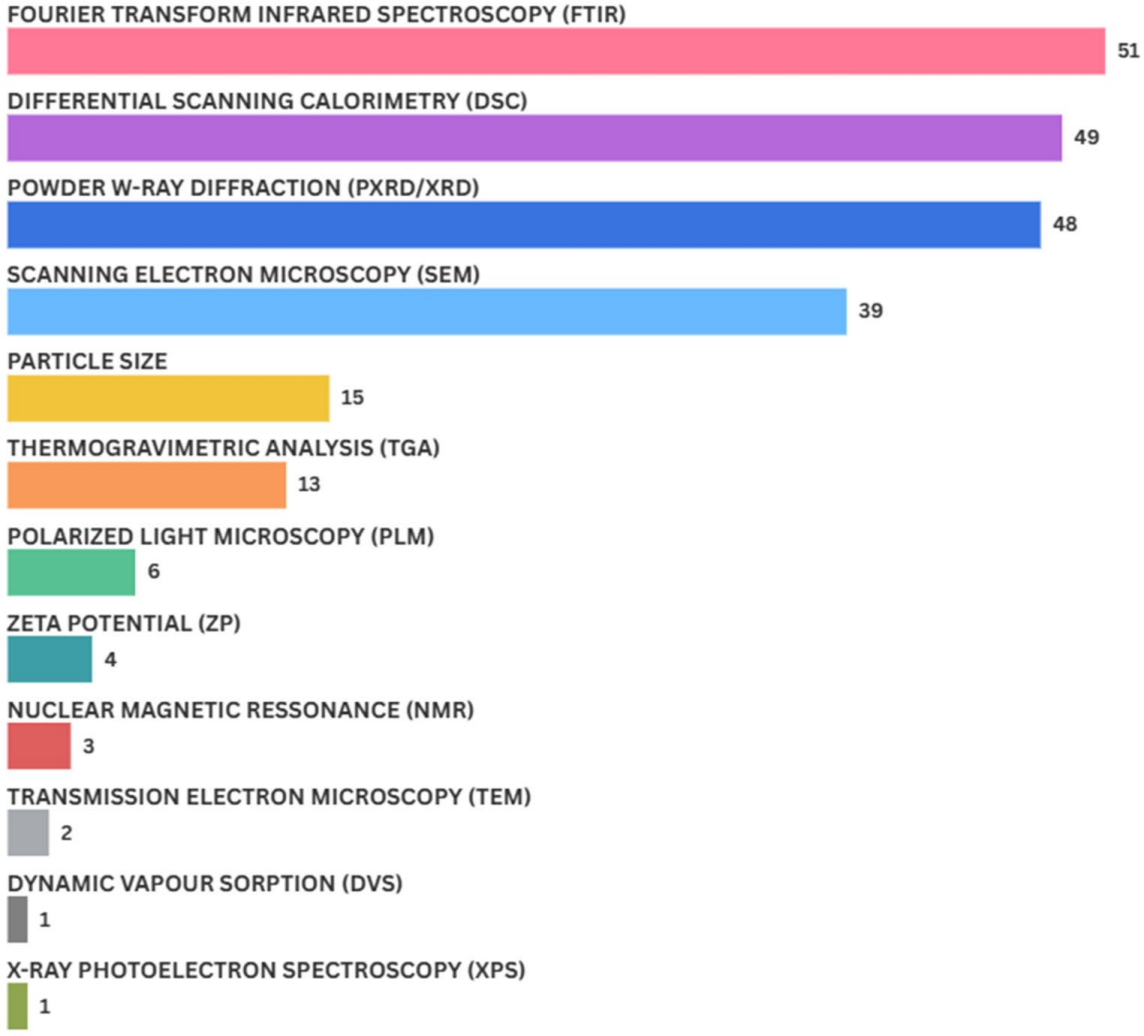
Representative graph of the characterization techniques employed

Because of their complex composition, the characterization and quality control of ASDs represent considerable challenges. To distinguish these systems from solid solutions, analytical methods capable of assessing compound crystallinity are necessary, with ASDs being defined by the absence of crystalline order [116]. The physicochemical characterization techniques most frequently reported in the reviewed studies are summarized in Fig. 4.

## Discussion

### Critical aspects of plant-derived apis in amorphous solid dispersions

Amorphous solid dispersions (ASDs) represent a promising strategy to overcome the intrinsic limitations of plant-derived active pharmaceutical ingredients (APIs), which often exhibit low aqueous solubility, low permeability, chemical instability, and rapid metabolism. Across different phytochemical classes, ASDs consistently promote increased dissolution rates and, in many cases, improved pharmacokinetic and pharmacodynamic outcomes. However, a more detailed analysis of the literature reveals that these benefits are highly system-dependent and cannot be generalized across different formulations, carriers, or manufacturing routes. One of the most recurrent challenges for phytochemicals is their poor intrinsic solubility, which compromises dissolution and therapeutic efficacy. Several studies have shown that amorphization can directly address this barrier. For instance, the major bioactive of ginger, 6-gingerol, displayed improved solubility and reduced IC_50_ in gastric adenocarcinoma cells after ASD formulation [34, 128]. Similarly, β-carotene isolated from *Pandanus conoideus* [27] baicalein [77] and oleanolic acid [90, 91] all exhibited marked increases in dissolution and bioavailability once incorporated into ASDs. In the case of resveratrol, Yu et al. [76] reported improved solubility with Soluplus and PVP K-30, though dissolution profiles varied depending on the polymer, highlighting the critical role of excipient selection.

Other pharmacokinetic barriers associated with these materials include low permeability, efflux transport, and extensive first-pass metabolism, for which amorphization strategies have also proven beneficial. Compounds such as berberine, despite their broad pharmacological potential, exhibit low bioavailability due to factors such as lipophilicity, P-gp-mediated efflux, and intense metabolism [63, 65, 129]. Ternary ASDs enhanced permeability in Caco-2 models and improved in vivo hypoglycemic activity [63, 65], while Guo et al. [64] observed reduced IC_50_ in colorectal cancer cells with an ASD containing Eudragit S100. Comparable benefits were reported for artemisinin [89], cannabidiol (CBD) [83, 84], naringenin [87], and diosgenin [92], with formulations achieving superior absorption, improved dissolution, and in some cases, extended half-life. Taken together, these findings suggest that ASDs may partially overcome complex absorption limitations, although these benefits are system-dependent and cannot be generalized to all formulations, polymers, or manufacturing methods.

Amorphization has also been applied to traditional herbal preparations, modernizing their pharmacological use. Extracts from *Tripterygium wilfordii*, rich in triptolide and celastrol, were reformulated as ASDs with controlled release, improving both solubility and antibacterial activity [32, 130]. In the case of *Citri Reticulatae Pericarpium* (CRP), ASD-based extraction methods increased yield while offering a simpler and more sustainable alternative to conventional techniques [11, 29], with subsequent studies showing enhanced antiepileptic activity in zebrafish [28]. Similarly, ASDs prepared from *Boerhaavia diffusa* root extract [22] and *Centella asiatica* glycosides [30] not only improved dissolution but also resulted in superior antioxidant and anti-ulcer effects in vivo. These cases highlight the role of ASDs in bridging traditional medicine and modern formulation science.

Beyond physicochemical improvements, several studies demonstrate that ASDs can potentiate biological activity, with solubility enhancement ultimately reflected in therapeutic outcomes. For example, silymarin dispersions increased dissolution up to five-fold, accompanied by improved anti-inflammatory and hepatoprotective outcomes [35, 36], while hecogenin acetate ASDs amplified analgesic effects in neuropathic pain models [85]. Curcumin, despite its broad bioactivity, suffers from poor bioavailability; multiple ASD strategies not only improved solubility but also yielded stronger antimicrobial, antioxidant, and anti-inflammatory effects [67–71]. Other notable examples include RA-XII, an antitumor compound from *Rubia yunnanensis*, where a NADES-based ASD enhanced oral solubility and cytotoxicity [88], and magnolol, which exhibited increased plasma exposure and improved antioxidant and growth-promoting effects in chickens after amorphization [86]. Collectively, these findings indicate that ASDs can extend beyond formulation-oriented improvements to deliver therapeutic gains.

In many cases, marked increases in solubility and dissolution rate do not translate into proportional improvements in pharmacological activity or systemic bioavailability. This dissociation is often attributed to the persistence of pharmacokinetic barriers, such as instability in the gastrointestinal tract, extensive first-pass metabolism, and interactions with efflux transporters. Widely studied compounds, such as berberine and resveratrol, exemplify these limitations, as even after amorphization they remain susceptible to rapid biotransformation and restricted intestinal absorption. In summary, a comparative analysis of the studies demonstrates that, although ASDs represent a promising and versatile platform for the modernization of plant-derived active pharmaceutical ingredients, their therapeutic success depends on a complex balance among multiple factors [5]. The intrinsic properties of the phytochemical, the rational selection of the polymeric carrier, the processing method, and the intended therapeutic objective act in an interdependent manner in determining overall performance. The absence of a universal system underscores the need for systematic approaches, including rigorous comparative studies and strategies guided by quality by design (QbD) principles, to identify critical quality attributes and relevant process parameters.

## Manufacturing strategies for amorphous solid dispersions

### Solvent-based techniques

The solvent-based methods have been the most frequently and extensively investigated approaches when it comes to incorporating plant derived APIs, which is in part related to laboratory convenience and historical precedents [131, 132]. Among those, solvent evaporation has been the most widely applied technique, yielding consistent improvements in solubility, permeability, dissolution, and bioavailability for compounds such as berberine [63–65], resveratrol [76], curcumin [12, 68–70, 74, 75, 89], RA-XII [88], glycyrrhetic acid [93], baicalein [77], rutin [94], chebulinic acid [99], MT-102 [42], ellagic acid [105], linarin [97] and magnolol [86]. Beyond isolated compounds, this approach has also proven successful in generating stable ASDs from complex plant extracts, including silymarin [35, 36], boswellic acids [62], breviscapine [39] and crude extracts of *P. conoideus* [27], *Z. officinale* [34], *B. diffusa* [22], *S. miltiorrhiza* [41], *W. somnifera* [16], *G. biloba* [45], *P. longum* [52], *C. longa* [50], *O. tenuiflorum* [57], *K. parviflora* [60], TFE [48], TBESD [47], *F. indica* [59] and *C. asiatica* [30, 31].

Collectively, these articles indicate that solvent evaporation is a highly adaptable method to various phytochemicals, from isolated compounds to crude extracts. However, questions remain of whether it represents the most appropriate approach, explaining the growth in interest in alternative methods. Furthermore, directly comparative studies reveal challenges to its selection, as observed by Tambe and Pandita [62], whose study showed that kneading outperformed solvent evaporation in enhancing solubility and release of boswellic acids, exhibiting method-dependent performance of the ASDs, even when containing the same API. Tafu and Jideani [55] also found that solvent evaporation wasn’t the best option to produce *M. oleifera* solid dispersions, where it was observed that the freeze-dried ASD exhibited the best solubility.

As a more sustainable alternative, co-grinding has gained attention due to being suitable for thermolabile compounds [29, 120, 133]. Zhu et al. [29] successfully optimized co-grinding for *Citri Reticulatae* Pericarpium (CRP), reporting enhanced extraction yields, while Onoue et al. [78] achieved stable dispersions with improved biopharmaceutical and hepatoprotective properties and Rani et al. [56] produced a *M. oleifera* ASD, which displayed better particle size and reduced moisture, as well as improving solubility. Although these studies display favorable outcomes, it’s important to note that no head-to-head comparisons were made, limiting conclusions about the methods performance relative to other techniques. Kneading also represents a sustainable and industrially appealing method, with the ability to produce dispersions of satisfactory homogeneity [123]. This technique was employed for hecogenin acetate (HA), resulting in ASDs with improved analgesic activity in neuropathic pain models [85] and *O. europaea*, producing a formulation with improved solubility and dissolution profile [43].

Spray drying, on the other hand, is one of the most established and versatile techniques, it’s successful applications with plant APIs are widely reported, with improved solubility and stability observed for compounds such as polypeptide-k, phloretin, artemether, diosmin, chrysin, paclitaxel myricetin, curcumin, and extracts from CRP [28, 67, 74, 81, 96, 98, 102, 104, 106, 107]. Although not involving plant-derived APIs, comparative studies reveal that spray-dried ASDs tend to achieve higher dissolution rates than HME formulations due to smaller particle size and higher surface area, but demonstrate inferior physical stability against recrystallization [134, 135], which should be a factor in the method selection.

Freeze-drying provides an attractive option for thermolabile compounds, operating through freezing, primary drying, and secondary drying to generate amorphous systems under vacuum [136]. It has been effectively applied to both isolated phytochemicals, such as neohesperidin [82], rutin [95], capsanthin [58] and diosgenin [92], and crude extracts, such as those from *T. wilfordii* [32], *G. glabra* [61], *P. lobata* [51] and *R. Paeonia* [53], exhibiting superior solubility when compared to solvent evaporation, microwave irradiation and melting [55].

### Melt-based techniques

As for melt-based methods, there’s been less exploration due to being constrained by their incompatibility with thermosensitive materials and reliance on miscibility in the molten state [80]. Melting techniques successfully improved CBD uniformity and tablet manufacturability [83], while quercetin dispersions achieved initial dissolution enhancement but suffered from phase separation between PEG and the flavonoid [79], highlighting the possibility of unpredictable outcomes even when using well established excipients/carriers.

For Hot-melt extrusion (HME), which has emerged as a particularly promising solvent-free, continuous process, capable of producing ASDs for both immediate and controlled release with superior powder flow compared to spray drying [114, 125–127], positive outcomes have been demonstrated for crude extracts of species such as *T. wilfordii* [33], *P. cuspidati* [49, 54] and *G. biloba* [46] as well as isolated compounds like oleanolic acid [91], curcumin [73], resveratrol [44], hesperidin [100] and naringenin [87], achieving markedly improved solubility and oral absorption through extrusion-based dispersions. Moreover, HME’s scalability is outstanding, dominating the current commercially available ASD market al.ong with spray drying.

### Novel techniques

Finally, emerging methods observed in recent years include some less conventional approaches, like microwave-assisted processing, which was successfully applied by Liu et al. [92] to diosgenin, producing ASDs with improved plasma concentration and retention time. Microfluidization, although rarely reported, enables simultaneous micronization and amorphization, as observed in the curcumin nanocomplexes developed by Zhang et al. [71], which exhibited enhanced solubility and thermal stability. Fluid-bed coating is a rarely described method where the API and carrier are dissolved in an organic solvent and subsequently sprayed onto fluidized solid particles [38]. Xie et al. [38] utilized this emerging technique to prepare silymarin solid dispersions, attaining quick dissolution and controlled release of several of the components present in this crude API.

Spray freeze drying (SFD) has also been explored, where atomized droplets are directly sprayed into a cryogenic liquid, reducing drying time and preventing heat-induced degradation [117]. The oleanolic acid ASD produced by Tong et al. [90] demonstrated stability, better dissolution and absorption when compared to commercially available tablets. Jet milling, another particle-size reduction approach, can increase surface area and exposure of functional groups, leading to enhanced dissolution. Li et al. [84] demonstrated that jet-milled CBD ASDs exhibited smaller particle sizes, higher solubility, and superior antioxidant and antitumor activities compared with spray-dried formulations, though uniformity and scalability remain significant challenges.

Finally, co-precipitation has been highlighted as a highly efficient method for producing homogeneous amorphous systems with complete amorphization of the active compound [115]. Comparative work by Liu et al. [92] demonstrated that co-precipitation outperformed microwave and freeze-drying methods, achieving superior in vitro solubility and in vivo bioavailability. The co-precipitation method was also successful in the production of curcumin ASDs [72]. While promising, these novel methods demonstrate a lack of scalability evidence, which requires further studies before industrial adoption due to the possibility of facing uniformity and performance challenges on a larger scale.

Therefore, the choice of production method directly impacts not only the physicochemical stability and dissolution performance of plant-derived ASDs but also their scalability and industrial applicability. While traditional methods such as solvent evaporation and spray drying remain dominant. Emerging approaches, including microwave processing, microfluidization, and co-precipitation, highlight the ongoing evolution of ASD technologies to meet the challenges posed by complex phytochemicals. Equally critical to the success of these systems is the selection of suitable polymers and carriers, whose interactions with plant-derived APIs ultimately define the stability, dissolution behavior, and therapeutic performance of the final formulation.

### Carriers and matrix formers for amorphous solid dispersions

The review has shown that biopolymers represent a minimal fraction of formulations despite their inherent advantages, with only three polymers, pea protein (PP), chitosan, and trehalose, being identified and each appearing in only a single study. Zhang et al. [71] selected PP due to its low allergenic potential, health benefits, resistance to gastrointestinal digestion, and strong affinity for hydrophobic compounds such as curcumin, in addition to being a sustainable approach.

Chitosan’s inclusion by Alves et al. [72] aimed to obtain a thermoresponsive hydrogel containing curcumin, while Kaur et al. [96] applied trehalose in spray-dried polypeptide-k dispersions. The limited adoption of biopolymers likely reflects concerns regarding batch-to-batch variability, moisture sensitivity, and less predictable physicochemical properties compared to synthetic alternatives, despite their biodegradability, biocompatibility, and alignment with sustainable pharmaceutical development [137, 138],

The semisynthetic polymers where more explored, with hydroxypropyl methylcellulose (HPMC) as the most frequently employed cellulose derivative. HPMC’s frequent use is related to its dual functionality as both a matrix former and precipitation inhibitor, with its amphiphilic character enabling hydrogen bonding with plant-derived APIs while maintaining supersaturation during dissolution [139–141]. This can be seen in the work of Wong et al. [67], where it was possible to stabilize the amorphous state of nanocurcumin while preventing recrystallization under supersaturate conditions.

HPMCAS, used by Cao et al. [86] to prevent recrystallization leveraging its stability and solubilizing capacity, is an enteric derivative, providing pH-dependent solubilization which makes it particularly suitable for plant compounds requiring intestinal targeting [142]. As for other semisynthetic options, Onoue et al. [78] used HPC-SSL as the polymeric carrier in an ASD designed to improve the oral hepatoprotective activity of nobiletin. Li et al. [84] selected DM-βCD for its biocompatibility, high solubility, and ability to encapsulate hydrophobic molecules. HPC was employed by Shi et al. [65] because of its superior stability and reduced hygroscopicity compared with phosphatidylcholine, along with evidence supporting its efficiency in encapsulation and stabilization. Furthermore, carriers like Maltodextrin, carboxymethylcellulose (CMC), Avicel, croscarmellose sodium, and Gelucire, where also cited, each serving different functions such as complexation, bulking, disintegration, or lipid-based solubilization [143, 144].

Synthetic polymers remain the most frequently used carriers, with Soluplus, PVP variants, and PEG being the most common. PVP K-30 represents the single most frequently employed carrier, reflecting its established track record in pharmaceutical development, while different PVP grades (K-25, K-30, VA 64, etc.) allow for molecular weight optimization to balance processing requirements with dissolution performance [140]. Liu et al. [88] prepared binary dispersions with PVP K-30 and RA-XII and ternary systems incorporating a natural deep eutectic solvent (NADES, betaine and mandelic acid). The NADES-containing formulations improved solubility, uniformity, dissolution, and cytotoxicity. PVP K-30 was also used by Mureşan-Pop et al. [81], Cong et al. [39], Xie et al. [38], Kawoosa et al. [105], Sherikar et al. [36] and others, all reporting improvements in solubility, activity, and stability. PVP K-25, with its high Tg and low recrystallization tendency, was applied in ASDs [63, 79].

Soluplus, an amphiphilic copolymer also among the most recurrent choices. It offers distinct advantages, improving wettability, inhibiting recrystallization, and maintaining supersaturation of dispersed compounds [32, 36]. Its amphiphilic nature, thermal stability, low hygroscopicity, and high Tg further justify its widespread use. Studies by Sharma et al. [28], Liu et al. [92], Anwer et al. [107], Chhimwal et al. [106], Wang et al. [93] and others reported significant improvements in solubility, dissolution and bioactivity using Soluplus-based ASDs.

Poloxamers, particularly Poloxamer 407, have shown the ability to enhance the dissolution and bioavailability of hydrophobic compounds [69]. Tambe and Pandita [62] evaluated Poloxamer 407 and Poloxamer 188 in ASDs, obtaining stable dispersions with improved solubility, with kneading proving superior to solvent evaporation. Eudragit S100 was selected for its pH-dependent release properties, enabling colonic targeting and gastric protection [64], while Tung et al. [103] opted for Eudragit L100 as carrier for his weakly basic API.

PEG is another broadly used carrier, with its molecular weight strongly influencing performance [80]. Van Hecke and Benali [80] compared PEG 1000, PEG 4000, and PEG 6000, identifying PEG 1000 as the most effective. Otto et al. [79] employed PEG 400 and PEG 8000 in ternary systems, citing their biocompatibility, safety, and prior success in ASD formulations.

Many studies have compared multiple carriers to determine the most suitable option for specific APIs. Kaewkroek et al. [34] tested PVP K-30 and Eudragit EPO for ginger extract, observing higher solubility of 6-gingerol with PVP K-30 and an inverse relationship between polymer content and solubility. Wannasarit et al. [30] employed Eudragit EPO in ASDs of Centella asiatica, achieving increased solubility, stability, and sustained release. Yu et al. [76] compared ASDs prepared with Eudragit EPO, PEG 6000, PVP K-30, and Soluplus for resveratrol. PVP K-30 and Soluplus offered the best physical stability but reduced dissolution due to strong interactions, while PEG 6000 improved dissolution but lacked stability. Eudragit EPO provided a balance between dissolution and stability, emerging as the best option.

Other comparative studies have also yielded important insights, Wong et al. [67] assessed PVP K-30, PEG 6000, and Poloxamer 188 in ternary ASDs with berberine and sodium caprate, concluding that PEG 6000 was most effective; Gao et al. [91] optimized oleanolic acid formulations with Soluplus, PVP-VA 64, and PEG 6000, finding PVP-VA 64 to yield the best dissolution performance; Ishtiaq et al. [70] tested Soluplus, Syloid XDP 3150, Syloid 244, and Poloxamer 188 in ternary curcumin ASDs, with Soluplus achieving the highest solubility; Koch et al. [83] compared colloidal silica, Neusilin US2, and Syloid XDP in CBD formulations, selecting Syloid XDP at 40% for superior performance; Moreira et al. [85] screened HPMC, PVP K-30, Soluplus, PEG 6000, and PVP-VA 64 in hecogenin acetate ASDs, concluding that only HPMC enabled successful kneading-based dispersions; and Bhalodiya et al. [22] compared PVP K-30, HPMC, and HPMCAS, reporting all stable at a 1:4 ratio, with PVP K-30 and HPMCAS achieving > 90% release in 3 h.

Additional contributions include Fitri et al. [27] who evaluated PVP K-30, PEG 4000, and PEG 6000 in ASDs, with PEG 6000 outperforming others in solubility; Zhaojie et al. [63], who compared PVP K-30, PEG 6000, and Poloxamer 188 for berberine, finding PEG 6000 plus sodium caprate superior; Mohylyuk et al. [35], who compared Syloid XDP and Avicel PH-102 for silymarin, showing higher dissolution with Syloid XDP, while Tween 80 further improved performance with Avicel-based systems; and Yusuf et al. [68], who screened ASDs with Poloxamer 407, Poloxamer 188, HPMC, maltodextrin, and CMC, concluding that HPMC and CMC achieved superior dissolution, with HPMC showing the most favorable physical properties.

Overall, the evidence shows that the performance of ASDs is strongly influenced by the choice of carrier or matrix former. While synthetic polymers such as PVP, PEG, Soluplus, and Eudragit remain the most widely employed due to their versatility, stability, and capacity to maintain supersaturation, semi-synthetic and natural alternatives have also demonstrated promising results, particularly when aligned with sustainability goals. Comparative studies emphasize that there is no universal carrier; instead, the optimal selection depends on the physicochemical profile of the plant-derived API, the intended release characteristics, and the stability requirements of the final dosage form. These considerations highlight the need for comprehensive physicochemical characterization to understand drug–carrier interactions, confirm amorphization, and ensure reproducibility.

### Mechanistic insights into solubility enhancement and stability in ASDs

Amorphous solid dispersions (ASDs) containing plant-derived active pharmaceutical ingredients (APIs) represent an advanced strategy to mitigate major biopharmaceutical barriers of botanical bioactives, particularly poor aqueous solubility and limited permeability. Unlike conventional single drug–polymer ASDs, plant-derived systems frequently involve multicomponent matrices (e.g., target marker compound(s), co-extracted secondary metabolites such as phenolics, terpenoids and alkaloids, and endogenous plant constituents including polysaccharides, proteins, tannins and lipids) [16, 43, 74]. This compositional heterogeneity creates a competitive interaction environment in which multiple compounds simultaneously interact with the carrier polymer and with each other, resulting in complex intermolecular networks shaping miscibility, phase behavior, and the free-energy landscape of the amorphous phase [10, 145].

At the molecular level, stabilization and performance of an ASD are strongly influenced by drug–polymer interactions (and, in botanical matrices, by bioactive–polymer and excipient–polymer interactions), including hydrogen bonding, hydrophobic association and electrostatic effects. These interactions can reduce molecular mobility and suppress nucleation, thereby delaying phase separation and recrystallization. However, because several extract constituents may compete for similar interaction sites on the polymer, the strength and distribution of these interactions can be heterogeneous, which helps explain why formulations with superficially similar compositions may exhibit distinct stability and dissolution behavior [146–148].

Physical stability is critically controlled by molecular mobility, for which the glass transition temperature (Tg) serves as an informative proxy. In multicomponent botanical ASDs, low-molecular-weight constituents (and residual moisture) may act as plasticizers, depressing Tg and increasing segmental mobility, which accelerates structural relaxation, phase separation, and recrystallization during storage. Conversely, rigid polyphenolic structures, high-Tg carriers, or macromolecular constituents may exert antiplasticizing effects, restricting mobility and improving kinetic stabilization. Accordingly, Tg in botanical ASDs should be interpreted as an emergent outcome of competing compositional effects, rather than as a simple binary drug–polymer parameter [149, 150].

These mechanistic features can be inferred from solid-state and spectroscopic signatures, which are most informative when interpreted as indicators of miscibility and mobility. For example, DSC-derived Tg behavior and the absence of melting endotherms, X-ray diffraction loss of long-range order, and Fourier transform infrared spectroscopy evidence of interaction-driven band shifts/broadening collectively support a mechanistic picture of amorphization and stabilization. In botanical ASDs, overlapping spectral contributions from multiple constituents further underscore that such readouts should be interpreted in combination, as they reflect a heterogeneous and dynamic interaction landscape rather than a single dominant drug–polymer pair [14, 15, 22, 27, 70, 80, 87, 149].

From a biopharmaceutical perspective, botanical ASDs frequently operate under multicomponent supersaturation kinetics. Upon hydration, several constituents dissolve at different rates, generating a dynamic and competitive supersaturation regime [10, 16, 28, 80, 150]. Rapid amorphous dissolution and polymer-assisted wetting can produce a pronounced “spring” effect; however, less soluble or aggregation-prone co-constituents may precipitate early and act as heterogeneous nucleation templates, potentially triggering recrystallization of the marker API and causing a premature collapse of supersaturation (“parachute” failure). Thus, sustained exposure-relevant performance depends not only on drug–polymer interactions, but also on the balance between co-dissolution, precipitation inhibition, polymer selection, and microstructural organization within the matrix [39, 43, 47, 74, 80, 87, 151, 152].

Finally, these mechanistic considerations support more rational ASD design for plant-derived APIs by guiding polymer choice (interaction capacity and Tg), moisture and plasticization control, processing conditions that favor single-phase amorphous matrices, and fit-for-purpose analytical workflows. In multicomponent botanical systems, multi-analyte monitoring (e.g., Liquid Chromatography-based methods) can be especially valuable to distinguish true polymer-mediated maintenance of supersaturation from transient solubilization or selective precipitation, thereby linking solid-state mechanisms to biopharmaceutical outcomes [92, 153].

### Techniques for the physicochemical characterization of amorphous solid dispersions

The physicochemical characterization of amorphous solid dispersions (ASDs) is essential to confirm amorphization, detect residual crystallinity, and assess molecular interactions and stability. In the studies involving plant-derived active pharmaceutical ingredients (APIs), different analytical techniques were employed, each providing complementary information that contributes to a comprehensive understanding of ASD performance.

Fourier-transform infrared spectroscopy (FTIR), the most widely applied method in the reviewed studies, is another common analytical tool for ASD characterization and quality control. This technique relies on the unique vibrational frequencies of chemical bonds and functional groups, which interact with specific infrared wavelengths [115]. The resulting spectra reveal changes in the position and intensity of absorption peaks, providing insights into chemical interactions between APIs and carriers, as well as potential modifications during processing [84]. Nevertheless, FTIR presents limitations, particularly when dealing with plant-derived APIs. For instance, Otto et al. [79] reported that overlapping broad hydroxyl bands hindered the identification of interactions between quercetin and PEG or water.

X-ray diffraction (XRD), another common technique, enables the assessment of solid-state features [82, 84]. In the context of ASDs, XRD is particularly useful to confirm whether the processing strategy achieved amorphization. The technique is based on directing filtered and collimated X-rays onto the sample, producing diffraction patterns according to the degree of crystallinity, as defined by Bragg’s law. XRD is considered the gold standard for distinguishing crystalline from amorphous phases in a non-destructive manner, although it may have limited interaction with lighter elements [115].

Differential scanning calorimetry (DSC) is employed to investigate the thermal behavior of ASDs, highlighting transitions such as glass transition, melting, or recrystallization, and providing information about polymer–drug interactions [115]. It is frequently used as a complementary technique to XRD for confirming solid-state transitions [89]. Thermogravimetric analysis (TGA) also provides valuable data regarding thermal stability, residual solvents, and moisture content in ASD systems [154].

Nuclear magnetic resonance (NMR) spectroscopy has been used to probe molecular interactions in ASDs, complementing FTIR findings. Yu et al. [76] observed hydrogen bonding between plant-derived APIs and carriers such as PVP K-30 and Soluplus, whereas Eudragit EPO and PEG 6000 did not exhibit such interactions. In contrast, Liu et al. [88] reported negligible shifts in NMR signals, suggesting weak or absent intermolecular bonds.

Microscopy-based techniques also play an important role. Scanning electron microscopy (SEM) provides morphological and topographic details of ASD particles, although it requires sophisticated infrastructure and is relatively costly [115]. Polarized light microscopy (PLM) enables rapid detection of residual crystals through birefringence, offering sensitivity comparable to XRD [149]. For example, Mureşan-Pop et al. [81] confirmed crystallization in specific formulations by PLM, corroborated by XRD results.

Less frequently, transmission electron microscopy (TEM) has been employed to visualize crystallinity at higher resolution, though it may induce particle aggregation due to electron beam exposure [155]. In one case, Silva de Sá et al. [69] confirmed amorphous morphology with minor particle agglomeration. Dynamic vapor sorption (DVS) was applied by Van Hecke and Benali [80] to assess hygroscopicity, showing that quercetin–PEG dispersions exhibited reduced hydrophobicity compared to isolated quercetin. X-ray photoelectron spectroscopy (XPS), as reported by Tong et al. [90], was further used to determine surface distribution of polymers in ASDs, identifying recrystallization phenomena in the presence of sodium caprate.

Taken together, the application of these complementary analytical methods demonstrates that robust physicochemical characterization is indispensable for ASDs, mainly containing plant-derived APIs. By integrating thermal, spectroscopic, diffraction, and microscopic data, researchers can confirm amorphization, elucidate drug–carrier interactions, and anticipate stability or performance issues. This comprehensive approach ensures not only scientific validation of the systems developed but also provides essential evidence to support their progression toward pharmaceutical application.

### Industrial perspective: Scalability, QbD considerations, and regulatory implications

From an industrial perspective, the feasibility of amorphous solid dispersions (ASDs) depends on selecting manufacturing routes that are scalable, controllable, and compatible with Good Manufacturing Practices (GMP) [131]. While several laboratory methods can generate amorphous dispersions, many are not readily transferable to industrial environments due to limited process control and difficulties in equipment qualification/validation and hygienic design under GMP. In contrast, spray drying and hot-melt extrusion (HME) are consistently highlighted as industrially relevant ASD technologies because they support scale-up and systematic control strategies, including continuous or hybrid production concepts [10, 19, 156, 157].

For plant-derived APIs, industrialization is further complicated by the intrinsic variability of botanical raw materials and extracts. A practical industrial prerequisite is therefore to build “API reproducibility” upstream, through botanical identity control, traceability, and extract standardization, before transferring ASD processing conditions. In this context, the industry commonly recognizes different extract control strategies (e.g., standardized extracts, quantified extracts, and “other” extracts controlled by analytical markers), reinforcing that botanical ASDs must be developed against *specification-driven* extract quality rather than a single-entity API paradigm [158].

Within this landscape, Quality by Design (QbD) should be positioned as the organizing framework for development and scale-up. Practically, QbD begins by defining the Quality Target Product Profile (QTPP) and then translating it into ASD-relevant Critical Quality Attributes (CQAs), including amorphous fraction, glass transition temperature (Tg), residual solvent (for spray drying), moisture sorption/hygroscopicity, particle size distribution, flow function/cohesion, compressibility indices, and stability/performance attributes (e.g., dissolution and maintenance of supersaturation) [115, 157]. For botanical ASDs, CQAs must also incorporate phytocomplex consistency, typically via marker quantification and/or chromatographic fingerprinting, because co-extracted constituents can materially influence stability and dissolution behavior [158].

Process Analytical Technology (PAT) operationalizes QbD by enabling real-time measurement and control of relevant material and process signals, an especially valuable capability when the strategic goal is continuous manufacturing. The FDA frames PAT as a system for “designing, analyzing, and controlling” manufacturing through timely measurements of critical quality and performance attributes, which aligns directly with ASD processes where real-time monitoring can prevent drift in solid-state properties and performance. In ASD manufacturing, PAT-relevant measurements may include in-line/at-line spectroscopy (e.g., NIR/Raman) for compositional/solid-state signatures, torque/pressure/temperature profiles in HME, and outlet temperature or residual-solvent proxies in spray drying, coupled to particle attributes that predict flowability and tableting performance. This approach supports stronger process capability, lower batch-to-batch variability, and more robust control strategies for continuous or hybrid lines [115, 157].

Regulatory expectations must be addressed explicitly because “botanical” does not exempt a product from demonstrating quality, safety, and (depending on classification) efficacy. In the United States, the FDA’s guidance for botanical drug development places strong emphasis on Chemistry, Manufacturing, and Controls (CMC) for complex mixtures, including control of raw materials, process consistency, and robust specifications appropriate to multicomponent preparations [159, 160]. In the European Union, EMA/HMPC guidance documents emphasize specification setting for herbal substances/preparations and the appropriate use of markers and reference standards for quality control of herbal medicinal products and traditional herbal medicinal products [161]. In Brazil, ANVISA similarly structures pathways for herbal medicines and traditional herbal products around identity, quality specifications, standardization/markers, and stability requirements, aligned with the product category [162].

Despite these advances, persistent limitations remain important for both industrialization and regulation: upstream variability (genetics, environment, harvest/post-harvest, extraction) that makes traceability and standardization non-negotiable; the challenge of defining and justifying marker strategies for multicomponent mixtures; ASD-intrinsic risks (moisture sensitivity, phase separation/recrystallization, performance drift); and practical constraints such as drug-loading limitations, which can be particularly consequential for extracts that require higher doses. Collectively, these points reinforce that successful industrial deployment of botanical ASDs requires scalable platforms (spray drying/HME) selected against thermal/solvent constraints, QbD with ASD- and extract-specific CQAs, PAT-enabled monitoring to sustain performance in continuous/hybrid manufacturing, and regulatory-ready specifications integrating marker-based standardization, fingerprinting, solid-state controls, and stability across the product lifecycle.

### Future perspectives

**Fig. 5:**
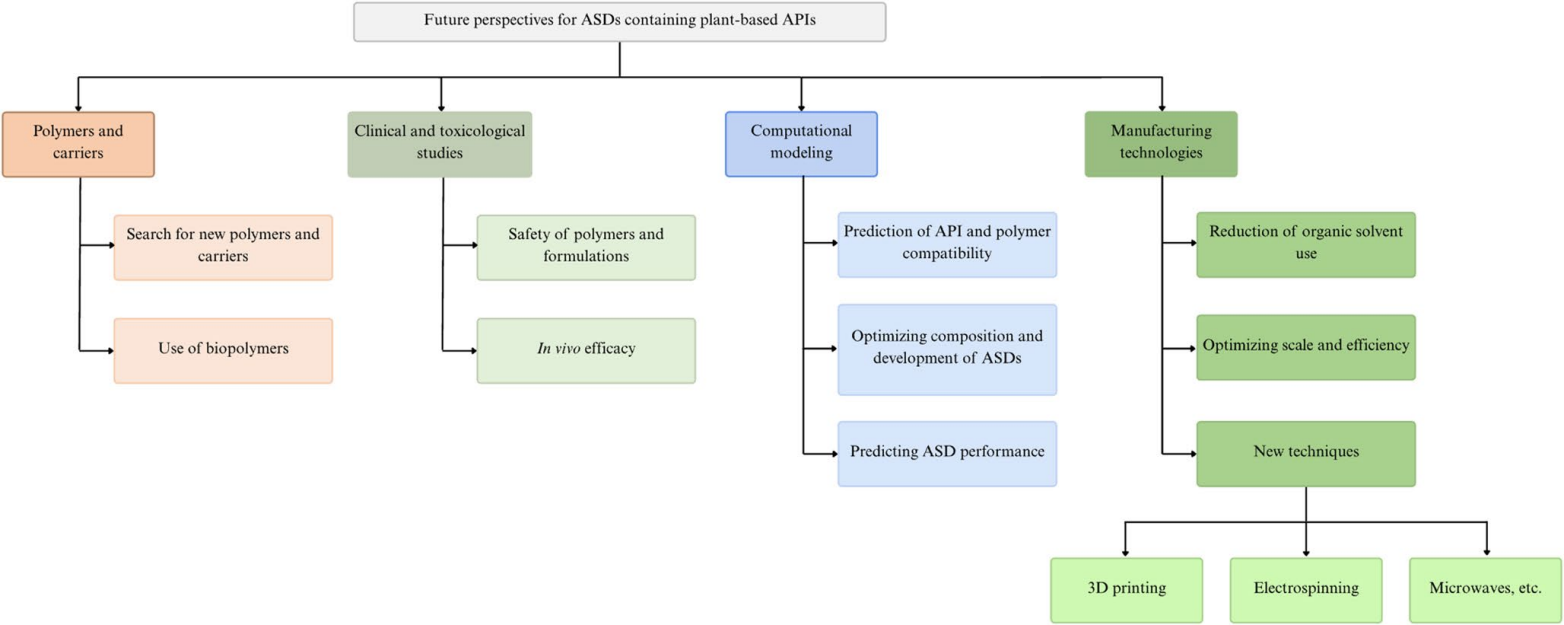
Flowchart outlining future perspectives for ASDs containing plant-derived APIs

As discussed, the use of ASDs to improve the bioavailability, bioactivity, and stability of active compounds represents a promising and increasingly explored field. Nevertheless, further investigations are required to consolidate this strategy for large-scale production and commercialization of formulations containing plant-derived APIs [163]. Figure 5 illustrates potential research directions for the advancement of this area.

The optimization of current manufacturing techniques, alongside the exploration of innovative approaches, remains a central challenge to ensure scalability, efficiency, and suitability for diverse physicochemical profiles of plant-derived compounds. While solvent evaporation continues to be applied, a clear methodological shift has been observed since 2020, with growing adoption of jet milling, freeze-drying, microfluidization, and hot-melt extrusion. This transition reflects not only technological advances but also the pursuit of sustainability, particularly through the reduction of organic solvent use. In addition, emerging technologies such as 3D printing, microwave-assisted processing, and electrospinning, establishing for synthetic drugs, offer opportunities for tailored ASD development with plant-derived APIs [164].

Carriers and excipients also constitute a key research frontier. The identification of novel polymers with desirable physicochemical properties could significantly enhance ASD stability and performance. In this context, biobased carriers present an attractive alternative, combining low toxicity and biodegradability with improved sustainability [164].

Finally, clinical and toxicological studies are essential to validate the safety and efficacy of both polymers and plant-derived actives in ASD formulations. Integrating computational modeling and predictive tools may further accelerate development, as demonstrated by Otto et al. [79] and Liu et al. [92], These in silico approaches enable the prediction of drug–carrier compatibility, formulation stability, and release behavior, thereby guiding rational design and reducing experimental costs [165]. Such strategies are expected to play a pivotal role in optimizing future ASD research and facilitating their translation into clinical and industrial applications.

## Conclusions

Amorphous solid dispersions (ASDs) have proven to be an effective strategy to overcome critical limitations of plant-derived active pharmaceutical ingredients (APIs), particularly poor solubility, restricted bioavailability, and physicochemical instability. Incorporation into hydrophilic polymeric matrices has been shown to generate stable amorphous systems that preserve and enhance the biological activity of compounds such as curcumin, quercetin, and several other phytochemicals. Despite these advances, challenges remain regarding industrial scalability and the refinement of manufacturing techniques, especially for the delivery of sensitive molecules. In this context, new approaches, including the use of biopolymers, the integration of computational modeling, and the application of emerging manufacturing technologies, represent promising alternatives to expand the applicability of ASDs. Furthermore, clinical and toxicological studies are indispensable to validate the safety and efficacy of these formulations, consolidating their pharmaceutical feasibility. Overall, ASDs stand out as a promising technological solution, with the potential to drive the development of more effective and stable herbal medicines, in line with the contemporary demands of the pharmaceutical industry.

## Data Availability

No datasets were generated or analysed during the current study.
